# Comparison of Healthcare Costs Associated With Patients Receiving Traditional Negative Pressure Wound Therapies in the Post-Acute Setting

**DOI:** 10.7759/cureus.11790

**Published:** 2020-11-30

**Authors:** Amy L Law, Blake Krebs, Bhavana Karnik, Leah Griffin

**Affiliations:** 1 Health Economics and Reimbursement, 3M, San Antonio, USA; 2 Life Sciences, Optum™, Eden Prairie, USA

**Keywords:** health economics, negative pressure wound therapy, chronic wounds, acute wounds

## Abstract

Objective

A retrospective national insurance claims database analysis evaluated total and wound-related costs for acute and chronic wound patients treated with negative pressure wound therapy (NPWT), comparing a product specific NPWT (NPWT-K) to other NPWT systems (NPWT-O).

Methods

Patients with one or more NPWT claims between January 2016 and September 2018 in an outpatient setting with continuous medical and pharmacy benefits for six months before the initial (index) NPWT claims and 12 months post index were assessed. The cohorts were propensity score matched based on age, gender, comorbidities, and payer type. Each cohort included 3,368 patients after matching. Costs were evaluated at 30 days, three months, and 12 months after initial NPWT placement. Hospital admission rates, emergency room (ER) visits, and NPWT device switching were evaluated at 30 days. Differences were analyzed by t-test and chi-square test.

Results

At 30 days, wound-related costs were $8,583 and $11,334, and total cost to treat was $17,809 and $24,405 for NPWT-K and NPWT-O, respectively (p < 0.0001). NPWT-O patients had higher NPWT, wound-related, and total costs across all time periods, as well as a longer average length of therapy (p = .0039). There was no statistical difference in 30-day hospital admissions (p = 0.089); although 30-day ER visits were higher for NPWT-K (4.9% vs. 3.3%, p = 0.0007). A higher degree of switching from NPWT-O to NPWT-K occurred at 30 days (NPWT-O 2.5% vs. 0.4%, p < 0.0001).

Conclusions

This comparative effectiveness analysis indicates differences remain across NPWT suppliers in wound-related and total cost to treat for patients who receive durable NPWT in the outpatient setting.

## Introduction

There is a growing understanding that wound care, although hard to monetize, is costly. Health expenditures in wound care have been estimated between $28.1 billion and $96.8 billion for the Medicare population alone. A national estimate of wound care would significantly exceed these expenditures [1]. Nearly 15% of Medicare beneficiaries have at least one type of wound or wound-related infection, with care largely occurring in the outpatient setting [1]. Treatment of these patients, who often have multiple comorbidities that impede wound healing, is a multi-disciplinary effort. Cross-collaboration between providers and suppliers across care settings can help improve the quality and effectiveness of care and benefit both the patients and the payers.

Negative pressure wound therapy (NPWT) is an advanced adjunctive therapy that has been used to facilitate healing in acute and chronic wounds [2-7]. Typically, providers prescribe NPWT for the most challenging wounds when granulation tissue formation cannot be achieved via other methods or when other wound care methods have been tried or considered and ruled out. There are numerous studies that have evaluated the cost effectiveness of NPWT versus advanced wound dressings in a variety of settings and wound types. These studies have shown that NPWT has been associated with fewer hospitalizations, emergent care incidents, complications, amputations, dressing changes, decreased personnel commitments, shorter hospitalization, and reduced treatment times [2,8-16]. A recent evidence assessment of NPWT by The Independent Institute for Quality and Efficiency in Health Care (IQWiG) in Germany found evidence in favor of NPWT for wound closure and hospital stay, as well as rehospitalization compared to standard wound therapy in wounds with secondary wound healing [17]. This technology assessment resulted in approval of NPWT for use in the community setting by The Federal Joint Committee (G-BA, Gemeinsamer Bundesausschuss in German) for all Germans with mandatory health insurance [18].

While the majority of the literature reports outcomes using one model of NPWT with reticulated open-cell foam dressings (V.A.C.® Therapy, KCI, now part of 3M Company, San Antonio, TX), the number of NPWT alternatives has continued to evolve; however, there have been few head-to-head comparisons. The varying costs of the different NPWT therapy systems raise the issue of comparative effectiveness. Additionally, despite continued wound care advocacy and educational efforts, policy-makers have failed to make advances in reimbursement payment methodologies to adequately reflect the support and service levels most complex wound patients require. To demonstrate differences in comparative cost and clinical effectiveness of NPWT suppliers, de-identified insurance claim data for patients receiving any model of NPWT were analyzed for costs, hospital admission, and NPWT device switching rates for the period following the initial NPWT claim in an outpatient setting.

## Materials and methods

Design and data source

A retrospective analysis was conducted using the large, longitudinal, closed system de-identified patient level database provided by Optum™ (Eden Prairie, MN).

All data were de-identified and accessed in compliance with Health Insurance Portability and Accountability Act privacy guidelines [19]. All data analyses were conducted by Optum™.

Patients

Patients were included if they had one or more diagnosis claims with an NPWT Healthcare Common Procedure Code (HCPCS E2402) in the outpatient setting between July 2015 and September 2019. Patients were grouped according to initial NPWT therapy received - NPWT-K (V.A.C.® Therapy, KCI, now part of 3M Company) and NPWT-O (other non-KCI models of NPWT). Claims data were analyzed at 30 days as well as three and 12 months after the index date of the first NPWT claim. Patients also had to have continuous medical and pharmacy benefits (commercial or Medicare Advantage) for six months pre-index and 12 months post index. Patients with another outpatient NPWT claim 12 months prior to the index claim were excluded.

Propensity score matching was performed for included patients on comorbidity, payer, age, and gender. NPWT-K patients were case-matched to NPWT-O, so no NPWT-O patients were excluded from the analysis.

Measures

Patient demographic and clinical characteristics were assessed on the index date. The Charlson comorbidity score was computed for the six-month pre-index period [20]. Total costs were evaluated at 30 days, three months, and 12 months, calculated as the sum of wound-related and non-wound-related services based on standardized costs, which removed variations in the cost of service based upon specific contractual arrangements between payers and providers.

Hospital admission rates were defined as the proportion of patients with a hospital admission within 30 days of the index claim. All non-pharmacy claims with a wound diagnosis within the top three diagnoses on the claim were categorized as wound-related; all pharmacy claims were categorized as non-wound-related. Wound types were classified according to the ICD-10-CM code on the claim. Switching to alternate NPWT was measured as the proportion of patients switching from the initial NPWT model to a different NPWT device within 30 days.

Statistical analysis

Cohort differences were assessed using t-test for treatment costs and chi-square test for admission rates. A p value < 0.05 was considered statistically significant. Data analysis was generated using SAS® software, Version 9.4 (SAS® Institute, Inc., Cary, NC) for Microsoft Windows® 7 (Microsoft®, Redmond, WA).

## Results

A total of 15,180 patients met the inclusion criteria (NPWT-K n = 11,812; NPWT-O n = 3,368). The analysis of the case-matched cohort included 3,368 patients in each group. Mean age was 67 years, and 50.5% of patients were male. The mean Charlson comorbidity score was 3.4 (median 3.0). Approximately 82% had managed Medicare insurance. The most common comorbid conditions were hypertension, diabetes without complications, hyperlipidemia, atherosclerotic heart disease of native coronary artery, and peripheral vascular disease (Table 1).

**Table 1:** Baseline demographic and clinical characteristics of the study population NPWT-K, product-specific negative pressure wound therapy; NPWT-O, other negative pressure wound therapy.

Baseline Characteristics	NPWT-K (n = 3,368)	NPWT-O (n = 3,368)
Mean age, years	66.9	67.0
Male gender, percent	50.6%	50.5%
Insurance		
Managed Medicare	81.9%	81.7%
Commercial	18.1%	18.3%
Charlson comorbidity	3.4	3.4
Top Comorbidities		
Primary (essential) hypertension	54.2%	52.7%
Type 2 diabetes mellitus without complications	28.9%	29.2%
Hyperlipidemia, unspecified	22.0%	22.9%
Atherosclerotic heart disease of native coronary artery without angina pectoris	17.0%	16.6%
Peripheral vascular disease, unspecified	12.8%	12.9%
Wound Mix		
Open wounds	25.1%	24.7%
Non-healing surgical	21.3%	15.6%
Diabetic ulcer	19.0%	18.7%
Pressure ulcer	9.5%	13.5%
All Other + Unknown	25.1%	27.3%

Diabetic ulcers (DFUs), pressure ulcers (PUs), open wounds, and non-healing surgical wounds represented almost 75% of wounds. NPWT-O patients had significantly higher (p < 0.0001) PU diagnosis and higher unknown wound diagnosis (no wound diagnosis listed on claim three months before and after the index NPWT application). NPWT-K patients had significantly higher non-healing surgical wound diagnoses (p < 0.0001).

Within the study population, wound-related and total cost to treat were significantly higher for the NPWT-O group across all time periods (p < 0.0001, Table 2). These statistically significant differences remained across all wound types (Table 3).

**Table 2:** Average wound-related and total cost to treat across time periods.

Cost Category	NPWT-K (n = 3,368)	NPWT-O (n = 3,368)	% Difference	p Value
30 Days				
Wound-related	$8,583	$11,334	32%	<0.0001
Total cost to treat	$17,809	$24,405	37%	<0.0001
Three Months				
Wound-related	$17,615	$23,919	36%	<0.0001
Total cost to treat	$40,827	$53,884	32%	<0.0001
12 Months				
Wound-related	$35,625	$48,640	37%	<0.0001
Total cost to treat	$105,844	$137,928	30%	<0.0001

**Table 3:** Average wound-related and total cost to treat across wound types at 30 days, three months, and 12 months NPWT-K, product-specific negative pressure wound therapy; NPWT-O, other negative pressure wound therapy.

Cost Category	NPWT-K	NPWT-O	% Difference	p Value
Pressure Ulcer	n = 320	n = 455		
30 Days				
Wound-related	$12,343	$15,385	25%	0.0204
Total cost to treat	$19,861	$26,475	33%	<0.0001
Three Months				
Wound-related	$27,217	$36,151	33%	0.0006
Total cost to treat	$47,588	$61,277	29%	0.0002
12 Months				
Wound-related	$70,326	$83,240	18%	0.0432
Total cost to treat	$133,514	$161,276	21%	0.0039
Diabetic Foot Ulcer	n = 640	n = 651		
30 Days				
Wound-related	$11,351	$16,102	42%	<0.0001
Total cost to treat	$20,593	$28,750	40%	<0.0001
Three Months				
Wound-related	$28,292	$39,400	39%	<0.0001
Total cost to treat	$53,915	$71,242	32%	<0.0001
12 Months				
Wound-related	$63,175	$90,342	43%	<0.0001
Total cost to treat	$147,753	$194,263	31%	<0.0001
Non-healing Surgical Wound	n = 717	n = 525		
30 Days				
Wound-related	3,650	6,931	90%	<0.0001
Total cost to treat	13,767	20,752	51%	<0.0001
Three Months				
Wound-related	6,784	11,226	65%	<0.0001
Total cost to treat	30,337	42,994	42%	<0.0001
12 Months				
Wound-related	11,085	17,154	55%	0.0013
Total cost to treat	77,924	104,135	34%	<0.0001
Open Wound	n = 844	n = 831		
30 Days				
Wound-related	$6,560	$8,096	23%	0.0064
Total cost to treat	$15,562	$22,074	42%	<0.0001
Three Months				
Wound-related	$10,794	$14,009	30%	0.0004
Total cost to treat	$33,538	$44,806	34%	<0.0001
12 Months				
Wound-related	$17,243	$23,276	35%	0.0004
Total cost to treat	$86,861	$112,168	29%	<0.0001

**Figure 1:**
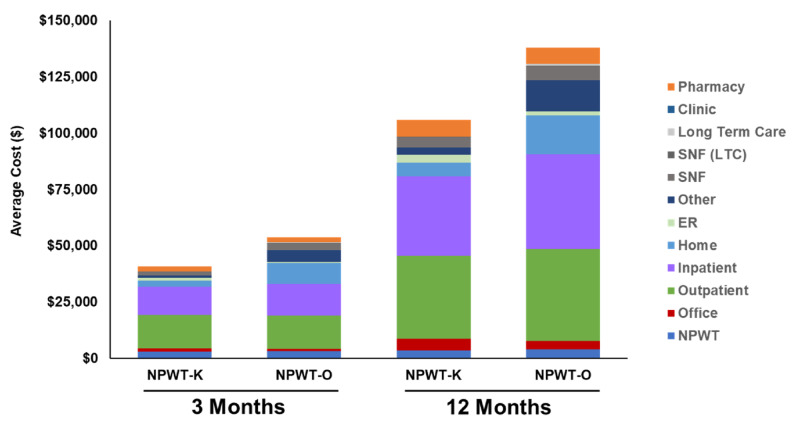
Total average cost to treat for three and 12 months NPWT-K, product-specific negative pressure wound therapy; NPWT-O, other negative pressure wound therapy.

NPWT-O wound-related expenditures were driven by statistically significantly higher NPWT, inpatient, home health care, skilled nursing facility, long-term care, and other expenses at three and 12 months. NPWT-K had higher office and emergency room (ER) costs (Figure 1).

NPWT-O patients had statistically higher NPWT cost at across all time periods, $107 higher (p < .008) at 30 days, $348 higher (p < .0001) at three months, and $408 higher (p < .0001) at 12 months. NPWT represented 6%-7% total cost to treat at three months and 3% of total cost to treat at 12 months. There was no statistical difference in 30-day hospital admissions (NPWT-K 15.2% and NPWT-O 13.7%, p = 0.089) although 30-day ER visits were higher for NPWT-K vs. NPWT-O (4.9% vs. 3.3%, p = 0.0007). There was a higher degree of switching from NPWT-O to NPWT-K at 30 days (NPWT-O 2.5% vs. 0.4%, p < .0001).

**Figure 2:**
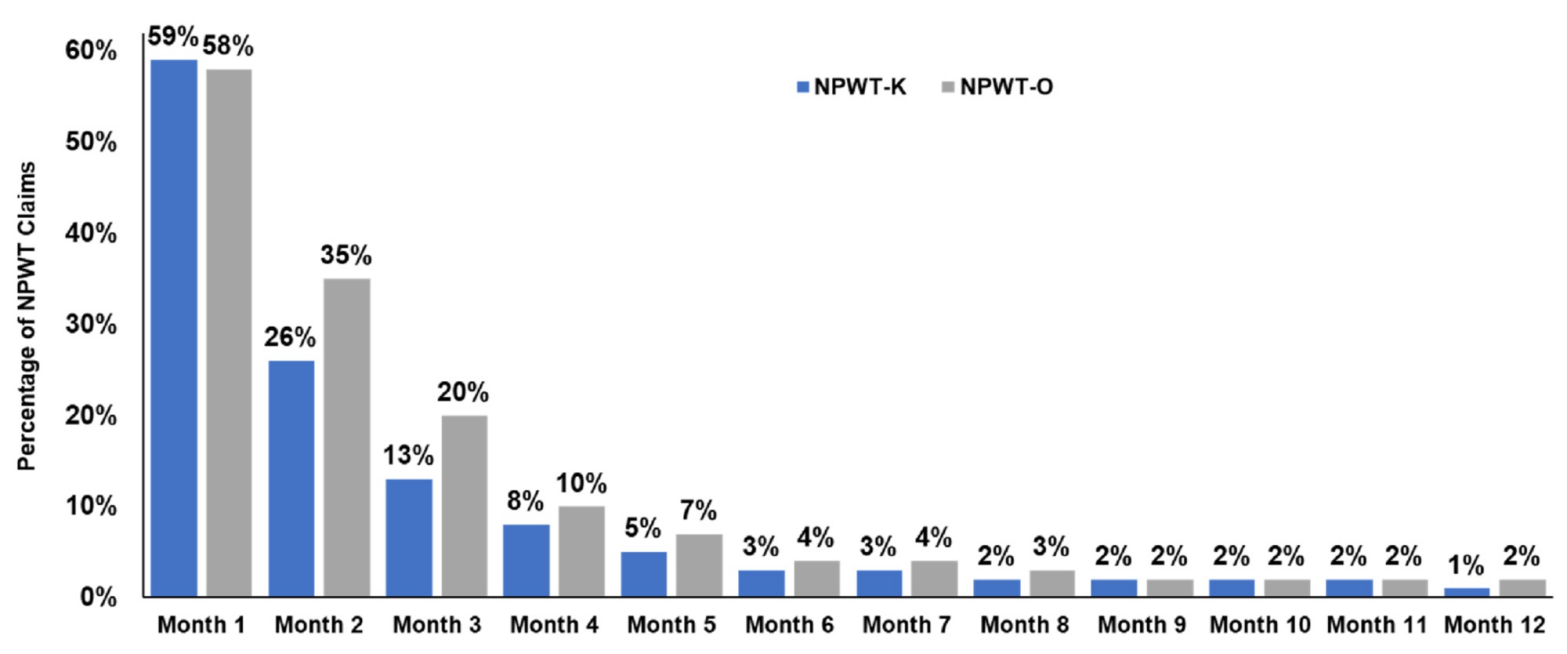
Total average cost to treat for three and 12 months NPWT-K, product-specific negative pressure wound therapy; NPWT-O, other negative pressure wound therapy.

More NPWT-O patients had an NPWT claim in each subsequent month following the initial outpatient claim, representing a longer average length of therapy; 20% of NPWT-O patients were still on therapy at month three versus 13% for NPWT-K (Figure 2).

## Discussion

Despite the growth in the use of traditional NPWT in the out-of-hospital setting, head-to-head studies have been lacking. Longitudinal analysis of real-world data allows us to capture the diversity of patients who utilize NPWT and compare costs across multiple sites of care, which would be cost-prohibitive in a randomized-controlled trial. Importantly, it also facilitates evaluation across a longer time horizon, which can be helpful comparing cost effectiveness for this complex patient population. Fife et al. highlighted the usefulness of real-world registry data in the 2012 analysis of 5,240 patients and 7,099 wounds (across all therapies) and noted an average time to heal of 15 weeks (107 days; SD: 150.29), with 10% of wounds taking 33 weeks or more to heal [21]. The authors noted that several factors can be defined that increase the duration and cost of wound care, including wound etiology as well as several specific patient factors including two or more comorbidities, as seen in our study population. A later study by Driver et al. found that chronic wound patients treated with NPWT took an average of 270 days to heal [22].

In this retrospective analysis we compared total costs and wound-related costs associated with use of NPWT-K and NPWT‑O to treat acute and chronic wounds in an outpatient setting. Despite a higher average therapy price for NPWT-K, NPWT-K patients had statistically lower NPWT therapy costs across all time periods, and fewer patients required an extended duration of therapy vs. NPWT-O. The NPWT-K group also had lower mean total costs and wound-related costs across all time periods. These total cost differentials support earlier findings of NPWT-K vs. NPWT-O [23]. These differences could be due to a myriad of factors not captured in claims analyses, such as differences in therapy effectiveness, patient adherence, communications with providers on wound progression, variances in care protocols, or other educational and support services provided by NPWT-K vs. NPWT-O. The statistically significant difference in switching from NPWT-O to NPWT-K may reflect that a preference for NPWT-K for any of these reasons exists. These variations also support the importance for purchasers and payers to look beyond therapy acquisition price for this advanced wound therapy to the bundle of services provided and associated economic outcomes.

This longitudinal analysis also supports more recent observations by Nussbaum et al. on the importance of focusing beyond the inpatient stay when assessing complex wound patients with comorbidities that impact healing [1]. Across all wound types, inpatient cost represented only 31%-33% of 12-month total cost to treat. Additionally, for the most complex patients who receive NPWT, traditional wound-related cost estimates may be low. In this cohort, wound-related expenditures commencing with NPWT placement in the outpatient setting accounted for $11,085-$23,276 in acute wounds and $70,236-$90,342 in chronic wounds in the following 12-month period, and this ignores costs associated with possible prior hospitalization. These costs were (not surprisingly) highest for the chronic DFU and PU patients where wound-related costs still represented 43%-53% of total patient healthcare expenditures at 12 months. These high wound-related costs at 12 months may reflect a low complete healing rate for chronic wounds, the prevalence of multiple wounds in these complex patients, or both.

Notably, little outpatient NPWT research is focused on acute wounds. Non-healing surgical wounds are estimated to occur in 3% of the Medicare population, and surgical wounds drive the highest total wound care cost to the system [1]. In this outpatient NPWT patient population, approximately 25% of patients had open wounds and up to 21% had non-healing surgical wounds. In this subset of patients, we saw $11,085-$17,154 wound care-related costs post discharge. This added post-surgical cost supports the growing use of NPWT prophylactically on surgical incisions in patients that are at risk of developing incision complications [24].

While there was no statistical difference in hospital admission rate post index claim, the high rate of a hospital admission within 30 days of an NPWT again supports the potential value of outpatient care coordination for patients with multiple comorbidities that impact wound healing. More recent advancements in care coordination services such as remote therapy monitoring (RTM) of NPWT patients suggest that patients benefit from outreach and education in the home; and payers have seen incremental savings of 26% for patients with NPWT RTM vs. NPWT without monitoring [25].

The cost findings of this study support prior research identifying the need for The Center for Medicare and Medicaid Services (CMS) and health policy-makers to include wound-relevant quality measures in all care settings as well as to develop quality measures that span the entire treatment period of the wound, chronic care models, and reimbursement models to drive better health outcomes and smarter spending in the wound care space [26]. Notably, the CMS focus on reducing the product acquisition cost of NPWT through its “Competitive Bidding” program for Durable Medical Equipment targets less than 3% of these complex wound patients’ wound-related expenditures. Importantly, these differences in total cost of care across suppliers also points to problems with the CMS program that only allows those suppliers with the lowest bid to be allowed to supply Medicare beneficiaries with NPWT.

Limitations

While analysis of total cost to treat can provide insights into comparative effectiveness, there are important limitations in the analyses of payer claims data. Claims could be missing or miscoded and might not provide proof positive of the presence of an actual condition. Patients in this study were required to have continuous medical and pharmacy coverage for at least 18 months; thus, results could be primarily applicable to patients in a stable, managed care setting. Perhaps more importantly, age of wound and size of wound can have a significant impact on healing time and may have differed between the cohorts. Also, the general nature of the data could demonstrate trends but could not provide information as to their specific causes.

We speculate that clinical and technical support services and training a may represent important differentiating factors when evaluating the comparative effectiveness of various brands of NPWT in the outpatient setting and may have played a role in the results of our study. NPWT-K has remained the market leader and has been used for over 20 years, which may provide a hidden confounder in the data.

Analysis of claims data on its own is limited due to a lack of a healing measure and key data elements that impact wound healing. However, it is a helpful tool for health plans, integrated delivery networks, Accountable Care Organizations (ACOs), and providers interested in managing total cost to treat across care settings. There is a growing acknowledgment of the importance of multi-discipline and multi-care setting coordination for wound patients who have underlying comorbidities that can impact healing. Selection of the right therapy may be only a small piece of the puzzle; the services and support of the supplier providing the therapy may be equally relevant in saving healthcare resources.

## Conclusions

The study supports the importance of focusing on outpatient costs for complex patients with wounds. Patients with wound diagnoses are costly, and for chronic wounds, much of the costs follow through at least 12 months of care. In this retrospective study, NPWT-K patients had lower total costs and total wound-related costs based on lower overall resource use across all time points assessed (30 days, three months, and 12 months). While costs for therapy use is a significant component of healthcare costs, reimbursement payment methodologies to reflect the support and service levels complex wound patients require may be equally important in reducing healthcare expenditures.
